# Calciphylaxis in the Setting of Alcoholic Cirrhosis: Case Report and Literature Review

**DOI:** 10.1177/2324709617710039

**Published:** 2017-05-24

**Authors:** Natasha Shah, Hafiz Muhammad Sharjeel Arshad, Yanxia Li, Rogelio Silva

**Affiliations:** 1 Advocate Christ Medical Center, Oak Lawn, IL, USA

**Keywords:** cirrhosis, calciphylaxis, alcohol

## Abstract

Calciphylaxis can be a severe life-threatening dermatologic disease that is a known complication associated with end-stage renal disease. However, multiple non-uremic etiologies that are not yet well studied can cause calciphylaxis. We report a rare care of a 40-year-old female with history of alcoholic cirrhosis without any evidence of renal dysfunction who presents with calciphylaxis.

## Background

Calciphylaxis can be a severe life-threatening dermatologic disease that is a known complication associated with end-stage renal disease.^1^ Calciphylaxis is characterized by skin and soft tissue necrosis. Necrotic ulcers develop secondary to calcification deposition and thrombosis of vascular arterioles. These ulcers can commonly occur on the lower extremities and can become gangrenous. The exact mechanism of this disease remains unclear. In patients with renal disease, the condition is associated with increased levels of parathyroid hormone, calcium, and phosphorous. Treatments are directed toward regulating these abnormalities.

However, this rare disease is now being seen in patients without any evidence of confounding renal disease. Multiple non-uremic causes of calciphylaxis have been reported in the literature including primary hyperthyroidism, malignancy, diabetes, protein C and S deficiency, vitamin D deficiency, Crohn’s disease, and alcoholic liver disease.^2^ Recent literature review suggests that, currently, there are fewer than 10 case reports that have been documented with calciphylaxis associated with alcoholic liver cirrhosis with or without concurrent renal disease.^1,2^

This article reports and highlights a rare case of calciphylaxis in the setting of alcoholic liver cirrhosis in the absence of renal dysfunction and reviews the current literature available on this rare but important topic.

## Case Report

**Figure 1.**
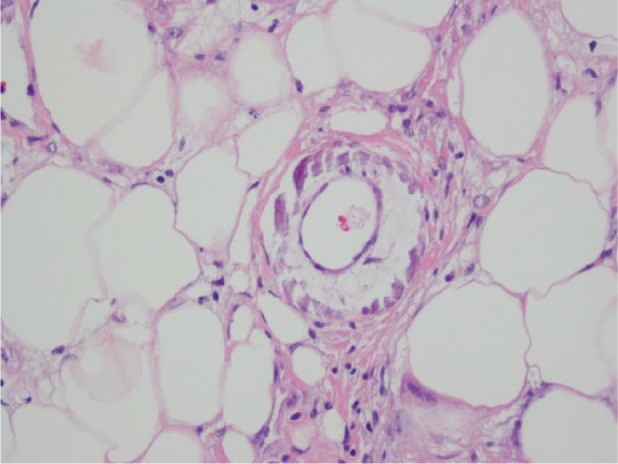
The skin lesion on the thigh was biopsied and it showed ulceration and subcutaneous tissue with fat necrosis.

**Figure 2.**
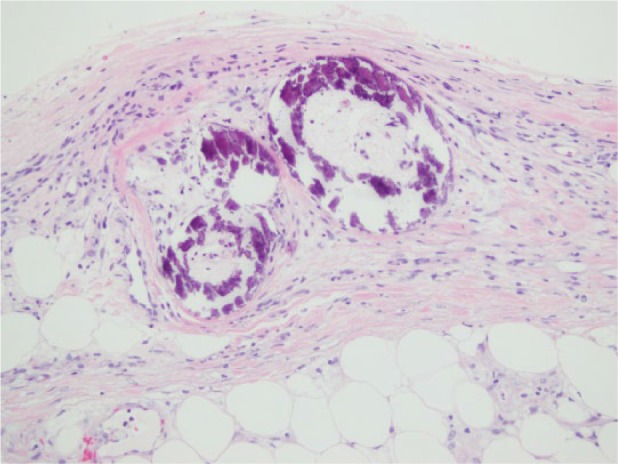
Multiple medium-sized vessels in the fat demonstrated prominent concentric calcification of the vascular wall, the characteristic pathological findings consistent with calciphylaxis.

A 40-year-old female with a history of cirrhosis secondary to progression of alcoholic hepatitis presented to the hospital complaining of painful nonhealing but worsening ulcerations on her thighs bilaterally. For the past several months, the patient has refrained from any type of alcohol abuse, which was represented by marked improvement in liver chemistries. The patient’s viral and autoimmune serology are negative. On physical exam, the patient presented with no clinical characteristics of decompensated cirrhosis such as jaundice, ascites, spider angioma, or encephalopathy. The patient did not have history of esophageal varices per esophagogastroduodenoscopy. The patient did have mild splenomegaly with spleen size of 13.4 cm that was revealed with computed tomography scan of the abdomen and hepatomegaly with the liver size of 19.5 cm. The lower extremities were remarkable for a blotchy erythematous pattern in the upper thighs resembling livedo reticularis. Extensive areas of eschar and skin necrosis were present, and the lesions appeared indurated and were extremely tender to touch. A biopsy of the skin and subcutaneous tissue of the ulcerations of the thigh were taken (Figure 1), and results showed small and medium-sized blood vessels with concentric calcifications (Figure 2). These findings are consistent with calciphylaxis.

The patient did not have any history of chronic kidney disease and did not present with any transient renal dysfunction. The patient had no history of hyperparathyroidism and calcium- or phosphorous-related disorders. Labs at the time of presentation were the following: creatinine 0.92 mg/dL (0.7-1.3 mg/dL), calcium 7.7 mg/dL (9-10.5 mg/dL), phosphorous 4.3 mg/dL (3-4.5 mg/dL), parathyroid hormone 35 pg/mL (10-65 pg/mL), alanine aminotransferase 11 units/L (0-35 units/L), aspartate aminotransferase 33 units/L (0-35 units/L), alkaline phosphatase 161 units/L (36-92 units/L), international normalized ratio 1.8, bilirubin 4.8, albumin 1.3 g/dL (3.5-5.5 g/dL), protein C activity decreased at 30%, and protein S activity decreased at 60%. The patient’s maximum calcium level during her admission was 9.8 mg/dL. Further autoimmune workup was sent and found to be unremarkable. This included the following results: ANA titer <80, anti-dsDNA = 10 unit/mL (abnormal >10 units/mL), anti-centromere antibody <0.2 AI (normal high >0.9 AI), cryglobulin not detected, ribonucleoprotein antibody = 0.4 (normal high = 0.9), Smith antibody <0.2 AI (normal high = 0.9 AI), Sjogren’s syndrome A antibody <0.2 (normal high = 0.9), Sjogren’s syndrome B antibody <0.2 (normal high = 0.9), smooth muscle antibody negative, rheumatoid factor IgM <0.5 (normal high >0.7), rheumatoid factor IgA <0.5 (normal high >0.7), Scl-70 antibody <0.2 (normal high = 0.9), myeloperoxidase antibody <0.2 (normal high >0.9), and serine protease 3 antibody <0.2 (normal high >0.9). These results were not suggestive of an underlying autoimmune disease.

She was started on intravenous (IV) vancomycin and IV piperacillin and tazobactam initially. On discharge she was given a 2-week course of ertapenem 1 g daily. She was also started on IV sodium thiosulfate 25%, 250 mg/mL injections 3 times a week, which has been shown to be beneficial in treatment calciphylaxis lesions.^3^ Extensive wound care was required including multiple debridement procedures and artificial skin grafting. Her wounds with treatment remained stable and mild improvement over the course of a month was seen. As she received care, she was evaluated for liver transplant.

## Discussion

Significant risk factors for calciphylaxis in addition to renal failure include hyperphosphatemia, elevated calcium levels, low serum albumin, high serum alkaline phosphatase, and female sex.^4^ Our patient’s case is remarkable as the patient developed calciphylaxis without any renal impairment and no electrolytes abnormalities. Secondary hypoparathyroidism can result secondary to renal disease, leading to increase in calcium and phosphorous level, which was not seen. The patient did have a low albumin secondary to cirrhosis causing low synthesis; therefore, the calcium level was corrected and it remained within normal range. It can by concluded that the patient’s development of calciphylaxis is likely due to alcohol-related liver disease. At this time, the mechanism is not very well understood. In patients with renal disease or hyperparathyroidism, a stressful environment can cause high levels of calcium and phosphorous, which leads to calcium deposition in vasculature leading to ulcerations and eventually necrosis. Sepsis, trauma, uncontrolled diabetes, or other clinical states can precipitate and lead to calciphylaxis in these patients.^5^

Liver dysfunction has been found to be correlated with low coagulation inhibitors, specifically protein C and S.^4,6,7^ In alcohol-induced liver disease–related calciphylaxis, few case reports in the literature have shown that deficiencies in protein C and protein S levels can result in vascular injury. Liver disease can lead to low synthesis of coagulation factors and other proteins that can lead to susceptibility to injury.^4^ In congenital diseases with deficient protein C and S levels, hemorrhagic necrosis of the skin and widespread vascular thrombosis results in death.^5^ Warfarin has been shown to decrease protein C and S level as well, which results in skin necrosis similar to what is clinically seen in calciphylaxis.^8^

Calciphylaxis is diagnosed by skin biopsy of the affected area. Though there are superimposed risks to performing a biopsy such as ulceration, infection, induction of necrosis, and bleeding, it is important to make the diagnosis as it can heavily influence treatment modalities.^9^ Three-phase technetium 99m methylene diphosphate bone scan has been used to make the diagnosis of calciphylaxis. A study showed sensitivity of 89% and specificity of 97%. However, data are limited.^10^ All medical therapies have been directed toward addressing the elevated calcium and phosphorous levels. In calciphylaxis secondary to non-uremic etiologies, no affective treatment is available as the pathology is yet not well understood. Supportive treatments included wound care, pain control, and empiric antibiotics, a regimen that our patient received.^2^ Kidney transplantation and parathyroidectomies have shown complete resolution of wounds in patients with renal disease or elevated parathyroid hormone levels in patients who were unresponsive to medical management.^11,12^ No literature is available at this time to determine if a liver transplant in cirrhotic patients leads to complete resolution of the dermatologic disease. In cirrhotic patients, with no renal disease, one case in the literature reported resolution of the wounds after conservative therapy including topical treatments and therapy directed at improving hepatic dysfunction with the use of lactulose, diuretics, and reduced sodium and phosphate intake.^13^ The use of corticosteroids has not shown any mortality benefits.^2^

## Conclusion

In conclusion, we report a rare care of calciphylaxis in the setting of alcoholic liver disease that we believe is likely in the setting of low protein C and S levels along with low albumin levels, which does not allow for proper healing. At this time, further research and investigative studies need to be done to properly understand the relationship between liver disease and calciphylaxis. Understanding the mechanism can lead to advanced treatments, which is necessary as mortality related to calciphylaxis has been noted to be more than 50%.^2^ Further research also needs to be carried out with regard to progression of disease after resolved liver disease with liver transplantation. Our patient had stopped drinking for 6 to 7 months; however, her vascular disease failed to regress even though her liver disease did not continue to worsen. For now, considering the diagnosis of calciphylaxis in the setting of alcohol liver disease will avoid delayed diagnosis and extensive workup and quicker treatment therapy.
